# Identification of a gene expression signature associated with breast cancer survival and risk that improves clinical genomic platforms

**DOI:** 10.1093/bioadv/vbad037

**Published:** 2023-03-22

**Authors:** Santiago Bueno-Fortes, Alberto Berral-Gonzalez, José Manuel Sánchez-Santos, Manuel Martin-Merino, Javier De Las Rivas

**Affiliations:** Cancer Research Center (CiC-IMBCC, CSIC/USAL and IBSAL), Consejo Superior de Investigaciones Científicas (CSIC) and University of Salamanca (USAL), Salamanca 37007, Spain; Cancer Research Center (CiC-IMBCC, CSIC/USAL and IBSAL), Consejo Superior de Investigaciones Científicas (CSIC) and University of Salamanca (USAL), Salamanca 37007, Spain; Department of Statistics, University of Salamanca (USAL), Salamanca 37008, Spain; Department of Computer Science, Universidad Pontificia de Salamanca (UPSA), Salamanca 37002, Spain; Cancer Research Center (CiC-IMBCC, CSIC/USAL and IBSAL), Consejo Superior de Investigaciones Científicas (CSIC) and University of Salamanca (USAL), Salamanca 37007, Spain

## Abstract

**Motivation:**

Modern genomic technologies allow us to perform genome-wide analysis to find gene markers associated with the risk and survival in cancer patients. Accurate risk prediction and patient stratification based on robust gene signatures is a key path forward in personalized treatment and precision medicine. Several authors have proposed the identification of gene signatures to assign risk in patients with breast cancer (BRCA), and some of these signatures have been implemented within commercial platforms in the clinic, such as Oncotype and Prosigna. However, these platforms are black boxes in which the influence of selected genes as survival markers is unclear and where the risk scores provided cannot be clearly related to the standard clinicopathological tumor markers obtained by immunohistochemistry (IHC), which guide clinical and therapeutic decisions in breast cancer.

**Results:**

Here, we present a framework to discover a robust list of gene expression markers associated with survival that can be biologically interpreted in terms of the three main biomolecular factors (IHC clinical markers: ER, PR and HER2) that define clinical outcome in BRCA. To test and ensure the reproducibility of the results, we compiled and analyzed two independent datasets with a large number of tumor samples (1024 and 879) that include full genome-wide expression profiles and survival data. Using these two cohorts, we obtained a robust subset of gene survival markers that correlate well with the major IHC clinical markers used in breast cancer. The geneset of survival markers that we identify (which includes 34 genes) significantly improves the risk prediction provided by the genesets included in the commercial platforms: Oncotype (16 genes) and Prosigna (50 genes, i.e. PAM50). Furthermore, some of the genes identified have recently been proposed in the literature as new prognostic markers and may deserve more attention in current clinical trials to improve breast cancer risk prediction.

**Availability and implementation:**

All data integrated and analyzed in this research will be available on GitHub (https://github.com/jdelasrivas-lab/breastcancersurvsign), including the R scripts and protocols used for the analyses.

**Supplementary information:**

Supplementary data are available at *Bioinformatics Advances* online.

## 1 Introduction

Breast cancer (BRCA) is the most prevalent type of tumor in women in Europe with more than 500 000 cases diagnosed per year. The accurate diagnoses and treatment are critical to improve the disease survival. BRCA treatment is determined by a standard categorization in four groups of tumors considering three main clinical biomarkers (Saini *et al.*, 2011): the estrogen receptor (ER; protein encoded by gene ESR1); the progesterone receptor (PR; protein encoded by gene PGR); and the human epidermal growth factor receptor-2 (HER2; protein encoded by gene ERBB2). These three biomarkers are regularly analyzed in hospital clinicopathology units using immunohistochemistry (IHC). These biomolecular factors are also commonly used to facilitate categorization into four standard groups of breast cancer: Luminal A, Luminal B, HER2-enriched and triple negative. Some proliferation markers, such as AURKA or MKI67, are also usually measured in the pathology units to improve risk estimation and treatment. However, the determination of these clinical markers by IHC suffers from frequent errors or inaccuracies (Li *et al.*, 2010) and the groups provided are frequently too heterogeneous or intermediate in terms of categorical expression of ER, PR or HER2 (Bartlett *et al.*, 2016; Mertins *et al.*, 2016; Venet *et al.*, 2011).

Modern genomic medicine based on omics technologies provides a new approach to the study of cancer by detecting the state of thousands of genes simultaneously. Moreover, it renders a way to interrogate the role of any gene or gene product as a potential biomarker associated with patient risk, prognosis or outcome. Several authors have proposed gene signatures related to the prognosis and survival of breast cancer (Cun and Fröhlich, 2012; Manjang *et al.*, 2021). However, as cancer is a complex and heterogeneous disease, the consensus among them is quite small and they are frequently sample dependent (Mertins *et al.*, 2016). In this regard, some commercial platforms have been developed based on gene expression detection for the prognostic analysis and prediction of breast cancer risk, such as Oncotype (https://www.oncotypeiq.com/) and Prosigna (https://www.prosigna.com/), which are already available in the clinic. Despite this advance, the overlap between the gene signatures and the agreement between the risk predictions for each patient, provided by both platforms, is often very different or uneven (Bartlett *et al.*, 2016; Mertins *et al.*, 2016); and they function as a black box where decisions cannot be interpreted in terms of clinical phenotypes and do not show the correlation with defined biomolecular markers (Manjang *et al.*, 2021). Therefore, the discovery and validation of robust and reproducible survival markers associated with the tumor risk is a critical step to achieve better disease prognosis and treatment.

Several authors have addressed the instability and irreproducibility of gene expression-derived prognostic signatures in breast cancer (Bartlett *et al.*, 2016; Cun and Fröhlich, 2012; Ein-Dor *et al.*, 2005). Within this problem, some integrative approaches attempted to combine protein interactome network information with gene expression data to more accurately predict prognosis (Das *et al.*, 2015). Others are based on ensemble methods (Abeel *et al.*, 2010; Gong *et al.*, 2018), that try to reduce the variance of the estimator considering different feature selection algorithms or different preprocessing methods.

In this work, we follow the approach of ensemble methods, but focusing on the use of bootstrap techniques that help to reduce the dependency of the gene signature on a particular sample set. A relevant objective in this approach was that the survival markers obtained were biologically interpretable (i.e. were specific genes identified as biomarkers of prognosis), and were measurable in their association with the clinical factors and categories that characterize each tumor sample. Therefore, we propose a robust framework based on a bootstrap strategy to discover a set of reproducible survival markers that best correlate with the IHC clinical factors ER, PR and HER2 (that are determined in routine clinical practice in breast cancer).

The specific computational strategy and algorithms used are described in Section 2, and applied to two independent breast cancer datasets that integrate a large number of primary tumor samples (1024 samples and 879 samples). As a main result, we report that the proposed set of survival markers improves the risk prediction and the stratification of breast tumors provided by the genes included in the genomic platforms Oncotype and Prosigna.

## 2 Methods

### 2.1 Datasets description

Survival markers are often obtained from studies with small sample size. This favors the instability of the gene signatures derived. In this research, we have built two independent large datasets that integrate gene expression, survival and clinical data coming from several studies and public repositories. The first integrated dataset corresponds to a collection of data series of breast cancer tumor samples that include genome-wide expression data (obtained with Affymetrix high-density oligo microarrays) and survival data with clinical information about the tumors. All the studies integrated in this dataset have been retrieved from Gene Expression Omnibus (GEO; https://www.ncbi.nlm.nih.gov/geo/) and they include the following information: (i) Gene expression platform used: GeneChips from Affymetrix Human Genome U133A and U133Plus2.0; (ii) Overall survival (OS) data available: event time $t_{i}$ and status, censored δ=1 or not censored δ=0; (iii) In at least a large part of the samples of the cohort, we had the values for the standard BRCA clinical markers determined by IHC: ER, PR and HER2.

Table 1 shows the survival studies retrieved from GEO that have been integrated in our first dataset. This dataset, after normalization and data control, included 1024 BRCA tumor samples: 380 with information about the protein expression values determined by IHC of the 3 clinical breast cancer markers (ER, PR and HER2); and the rest, 644 samples, with only genome-wide expression data and survival data.

**Table 1.** Compilation of BRCA expression series and sources

GEO ID	Original *N*	Final *N*	Survival type	Year	Journal
GSE6532	87	87	RFS, DMFS	2007	*J. Clin. Oncol.*
GSE12276	204	204	MFS	2009	*Nature*
GSE19615	115	115	RFS, MFS	2010	*Nat. Med.*
GSE17907	55	39	MFS	2010	*BMC Cancer*
GSE20685	327	327	OS, MFS	2011	*BMC Cancer*
GSE21653	266	252	DFS	2011	*BRCA Res. Treat.*
Total	1054	1024			

As we are integrating samples obtained under different protocols and conditions, the gene expression signal is not directly comparable due to the batch effect. The batch effect arises when different samples cluster together just because they belong to the same study. To avoid this problem, we applied the frozen RMA (fRMA) normalization algorithm proposed by Irizarry’s group (McCall* et al.*, 2010). We found that although this algorithm outperforms other approaches it should be adapted for our particular problem. The fRMA algorithm considers an empirical vector that gives different weight to each batch adjusting the expression to be comparable for different datasets. Weight vectors provided by Irizarry’s group cannot be applied to our problem, because it does not meet the same requirements. To overcome this problem, we designed different approaches, changing the number of subsets for each series, and the number of samples for each subset. This problem is explained in more detail in a study previously published by our group (Martinez-Romero *et al.*, 2018). After an extensive exploration of the data and quality control of the gene expression signal in the different batches, we selected a randomized sample size of 5 for each mini-subset. The number of mini-subsets for each series varied depending on the number of samples as explained in Supplementary Table S1.

As indicated above, we also used for this study a second independent dataset of BRCA tumor samples with full genome-wide expression data (obtained by RNA-seq technology) and survival data. This set was used in the validation of the gene signature (i.e. the list of selected genes) that we identified as good markers of survival with our first dataset. This dataset was made up selecting samples from The Cancer Genome Atlas (GDC Data Portal, https://portal.gdc.cancer.gov/). After cleaning and filtering the data, we selected and built a normalized set of 879 samples.

### 2.2 Identification of survival markers associated with IHC clinical markers of BRCA

**Fig. 1.**
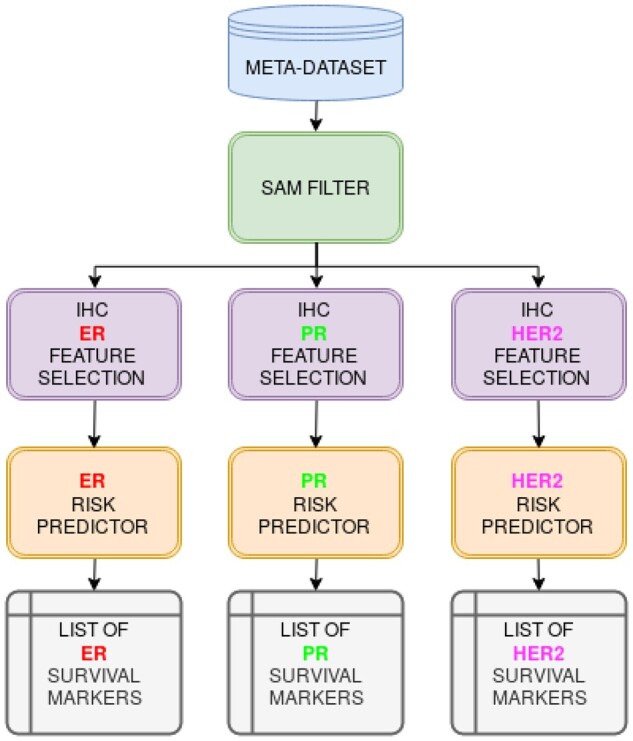
Diagram presenting a workflow with the steps to identify candidate ‘survival markers’ associated with risk prediction of the three standard IHC markers used in BRCA: ER, PR, HER2. The meta-dataset includes expression and survival data for 380 samples, with IHC levels known, used as ‘training set’; plus other 644 samples used as ‘test set’

In this section, we introduce a robust framework to discover a small but very significant subset of IHC-related genes that are also good markers of risk and survival in breast cancer. Figure 1 describes our approach. First, a robust differential expression algorithm (SAM) is applied to select the most variable genes and remove noisy features. Next, for each IHC marker (i.e. ER, PR and HER2, standard clinical markers in BRCA) a multivariate feature selection strategy is implemented based on an ensemble of linear classifiers (see Algorithm 1). The genes selected are those associated to the clinical variables with higher stability. Next, the gene markers for each clinical variable are ranked according to their ability to predict the risk and survival. To this aim, we have developed bootstrapped versions of univariate and multivariate feature selection algorithms based on Cox regression models. For each list, the genes more correlated to survival are selected giving rise to a subset of good survival markers associated to the standard clinical variables. Finally, the risk score is predicted based on the robust gene signature derived. An optimization algorithm is also introduced to stratify the patients in two risk groups. The following paragraphs describe each step of the framework in detail.

Algorithm 1 Robust feature selection algorithm based on bagging and the elastic-net classifier.

1: **Initialize:** Let G={0,1} be a clinical variable, *S* the whole set of patients, *X* the gene expression matrix and $N_{B}$ the number of resamples.

2: Train an ensemble of linear predictors for feature selection. Diversity is induced by bootstrapping

3: **for**j=1 to $N_{B}$**do**

4: Select randomly a subset of patients $S^{\left(j\right)}$ by sampling with replacement

5: Train a linear classifier via the elastic-net algorithm where the superscript denotes the *j*th bootstrap resample.

$$Y^{\left(j\right)}=\beta_{0}^{\left(j\right)}+{\left(\beta^{\left(j\right)}\right)}^{T}X^{\left(j\right)}$$

6: Select the subset of genes $g_{i}^{\left(j\right)}$ with non-null coefficient $\beta_{i}^{\left(j\right)}$. They are gene markers for the bootstrap resample $S^{\left(j\right)}$.

7: **end for**

8: For the ensemble of gene list compute the stability index $s_{i}=n_{g_{i}}/N_{B}$, where $n_{g_{i}}$ is the frequency of occurrence of gene *i*.

9: Rank genes and select the most stable ones.

10: Predict the clinical state using a voting strategy for the ensemble of classifiers.

11: **Return** the list of markers for the clinical variable and the IHC prediction.

The base of the multivariate feature selection strategy is the elastic-net algorithm proposed by (Friedman *et al.*, 2010). Although this algorithm is quite robust, some researchers (Das *et al.*, 2015) have pointed out that the genes selected in cancer transcriptomic studies are often quite unstable and sample dependent. To avoid this problem, we improve the robustness of our algorithm using a bagging strategy (Mbogning and Broët, 2016). An ensemble of linear classifiers is trained for feature selection and diversity among them is induced by bootstrap resampling. Several authors have applied ensemble methods in feature selection to improve the robustness of the gene markers found (Abeel *et al.*, 2010; Gong *et al.*, 2018; Zhao *et al.*, 2011). The ensemble of lists is combined by selecting the genes associated to the IHC variables with highest stability.

The elastic-net method is applied to the feature selection. The elastic-net is a linear logistic regression model with a regularization term that is between Lasso and Ridge Regression (Friedman *et al.*, 2010). This kind of predictor will shrink most of the coefficients to zero keeping only genes associated to the clinical variable. For simplicity, let *G* be the clinical variable that takes values in G={1,2}. The model can be extended easily for more than two categories. The logistic regression assumes: where Pr(G=i|x) is the a posteriori probability of class *i* and β is the vector of linear coefficients associated with the predictors. The model is adjusted by maximizing the regularized binomial likehood: $P_{\alpha }(\beta )$ is a regularization term that plays a critical role to avoid overfitting. For the elastic-net algorithm this term is defined as:


(1) $$\text{log}\;\frac{Pr(G=1|x)}{Pr(G=2|x)}=\beta_{0}+\beta^{T}x$$

(2) $$\underset{(\beta_{0},\beta )\in R^{n+1}}{\max}\frac{1}{N}\sum_{i=1}^{N}\{I(g_{i}=1)\;\text{log}\;p_{i}+I(g_{i}=2)\;\text{log}(1-p_{i})+\lambda P_{\alpha }(\beta )\}$$

(3) $$P_{\alpha }(\beta )=\sum_{j=1}^{p}\frac{1}{2}(1-\alpha )\beta_{j}^{2}+\alpha |\beta_{j}|$$

The α parameter determines the type of regularization considered and has a strong impact on the solution. Thus, for α=0, the model is equivalent to Ridge regression. The solution is dense and a large number of $\beta_{i}$ will become small but not equal to zero. For α=1, the model reduces to Lasso. The solution is sparse and most of the $\beta_{i}$ will become zero. The not null coefficients correspond to the predictor variables associated to the class label. When a subset of genes related to the clinical variable is slightly correlated, only one is chosen randomly. When α is between zero and one, most of the relevant genes related to the class variable are kept, although they may be slightly correlated among them. This improves the robustness of the subset of genes selected. Finally, λ is a regularization parameter that should be determined by nested cross-validation to avoid overfitting. Nested cross-validation implements a double loop of cross-validation. In the inner loop, the optimal regularization parameter is chosen to maximize the partial likehood deviation in log-scale. In the outer loop, the area under the receiver operating characteristic curve is estimated.

We have proposed a methodology to identify and select a set of genes related to the standard clinical variables in BRCA. Now, a relevant question for clinicians is which ones are significantly associated to the prognosis and survival of the patient. To address this, two complementary strategies are proposed. The first one is univariate and based on a robust version of the univariate Cox regression approach (Spirko-Burns and Devarajan, 2020). This strategy is quite robust to the particular training set of patients considered, but does not take into account the cooperative work among the genes, that is frequent to carry out a specific biological function. The second one is based on a multivariate Cox regression approach (https://rdrr.io/cran/uniCox/) proposed by (Tibshirani, 2009) and considers additive cooperation among the genes.

The univariate Cox proportional hazard regression algorithm is a linear technique that models the relative risk of patients. It does not assume a particular distribution for the hazard function and is less sensitive to the small sample size problem than other nonparametric approaches. Although the univariate Cox regression has been widely applied to gene selection and risk prediction in the literature, the stability and reproducibility of the list of genes obtained should be improved. To this aim, we have developed a bootstrapped version of the original Cox regression, following the same approach of Algorithm 1. In particular, an ensemble of univariate Cox regression models is trained for each gene. Diversity among the risk predictors is induced by bootstrapping. For each bootstrap sample *B*, a ranked list of genes is obtained considering a given metric $f_{i}^{\left(B\right)}$ for gene *i*. Different relevance metrics may be considered, such as the regression coefficient $\beta_{i}$, the log-rank statistic or the Wald statistic for the $\beta_{i}$. Finally, a summary statistic is computed along the ensemble of lists for each gene, based for instance on the median $f_{i}=\text{med}\{f_{i}^{\left(1\right)},f_{i}^{\left(2\right)},\ldots ,f_{i}^{B}\}$ (Abeel *et al.*, 2010).

Regarding the multivariate approach, we have considered the Cox proportional hazard regression model with $L_{1}$ norm penalty proposed by Tibshirani (2009). Let, $X_{1},X_{2},\ldots ,X_{p}$ be the expression levels of the *p* genes. For each sample *i*, let $\left(t_{i},\delta_{i}\right)$ be the survival time and the censoring indicator for patient *i* respectively. The hazard of death at time *t* given the observed values of the gene expression (λ(t|x)) can be modeled using a multivariate Cox regression model as: where $\lambda_{0}(t)$ is an unspecified baseline hazard function, $\left(\beta_{1},\beta_{2},\ldots ,\beta_{p}\right)$ are the regression coefficients and $\left(X_{1},X_{2},\ldots ,X_{p}\right)$ are the gene expression levels. $f(X)=\beta^{T}X$ is the linear risk score for the corresponding patient.


(4) $$\lambda (t|x)=\lambda_{0}(t)\;\text{exp}\;\left(\sum_{j}\beta_{j}X_{j}\right)=\lambda_{0}(t)\;\text{exp}\;\left(\beta^{T}X\right)$$

The $\beta_{j}$ coefficients are estimated by maximizing the partial log-likelihood with $L_{1}$ norm penalty: where $R_{k}$ is the set of patients at risk for time $t_{k}$ and λ is a regularization parameter that is estimated by 10-fold cross-validation. This parameter allows us to shrink most of the $\beta_{j}$ coefficients to zero selecting relevant risk markers.


(5) $$l(\beta )=\sum_{j=1}^{p}\sum_{k=1}^{n}\left(x_{kj}\beta_{j}-\text{log}\;\sum_{m\in R_{k}}\;\text{exp}\;(x_{mj}\beta_{j})\right)-\lambda \sum_{j}|\beta_{j}|$$

To improve the robustness of the multivariate Cox regression and the stability of the risk markers selected, a double-nested cross-validation is applied. The inner loop estimates the optimal λ regularization parameter for each predictor by 10-fold cross-validation and using a grid search strategy. In the outer loop, 10-fold cross-validation predicts the risk score considering nonoverlapping training and test sets. This strategy helps to avoid the overfitting, improving the risk prediction and the stability of the gene markers selected.

### 2.3 Accurate risk prediction and patient stratification

The risk prediction is carried out based on a small and robust subset of risk markers selected that correlate well with clinical variables. The risk curve is estimated for the training set using double-nested cross-validation and the multivariate Cox regression introduced earlier. Next, the patients in the training set can be stratified in two risk groups (low and high). The usual method for the risk stratification is based in a heuristic threshold such as the median. This strategy does not perform well when the groups of risk are unbalanced. In this article, the optimal threshold is estimated by maximization of the log-rank statistics. This method splits the patients in two risk groups that maximize the separability between the corresponding Kaplan–Meier curves. Below we provide a brief pseudocode outline that includes the steps of Algorithm 2 used for risk stratification.

Algorithm 2 Optimal risk stratification.

1: **Initialize:** Let *N* be the number of patients and $R_{j}$ the risk score for patient *j*.

2: Rank patients according to the risk score.

3: **for**j=1 to *N* **do**

4: Define threshold: $\theta_{j}=R_{j}$. Let *G* be the group variable defined as:

(6) $$G(j^{\prime })=\{\begin{array}{cc} 1 & R_{j^{\prime }}>\theta_{j}\\ 0 & R_{j^{\prime }}<\theta_{j} \end{array}$$

5: Compute the *P*-value using the log-rank statistic and the risk groups induced by *G*.

6: **end for**

7: Build the log-rank *P*-value curve versus the risk threshold value. The lowest *P*-value defines the optimal threshold $\gamma_{j}$.

8: Return risk threshold $\gamma_{j}$ and classify the patients in two groups

9: **End**

Once the Multivariate Cox Regression is trained, it can be applied to predict the risk score for a new sample to test. To do this, we need to provide for this new query sample the gene expression profile for the subset of gene markers selected. Following this protocol, a new sample to test (from a new patient) will be assigned to the low-risk group if the risk score is smaller than the optimal threshold estimated based on the training set. Conversely, it will assigned to the high-risk group if the risk score is larger than the optimal threshold.

## 3 Results

Survival studies in cancer rely frequently on tumor samples of small size. Therefore, the biological findings are sample dependent and cannot be generalized well. To avoid this problem, in this work the list of survival markers is obtained using a first dataset of 1024 tumor samples (described in Section 2.1 and collected and compiled for this research). We also use a second independent dataset of 879 tumor samples to validate the results. Survival data are available for all the samples in both datasets, and the clinical markers (ER, PR and HER2) have been determined by inmunohistochemistry for a subset of 380 patients of the first set. This subset has been used as the initial training set to identify the list of survival genes related to the clinical markers and to train a multivariate risk predictor. The rest of the 644 samples of the first set are used to validate the list of genes and to evaluate the risk prediction. Therefore, after selecting the candidate survival marker genes, we use the second independent cohort of 879 tumor samples for validation of the prognostic power of the genes (selected as survival markers) and for the final prediction of the risk assigned to each tumor. We have also performed done a comparison of the gene signature discovered in this work with the gene sets included in Oncotype and Prosigna/PAM50 clinical genomic platforms. Notice that Prosigna and PAM50 include the same list of 50 genes in their respective platforms (Wallden *et al.*, 2015). PAM50 is a well-known set of 50 genes that were found and identified by analyzing many genome-wide expression profiles of breast cancer patients (mainly by Charles M. Perou and collaborators), and included in a classifier of breast cancer tumors (i.e. PAM50) that defined five gene expression-based intrinsic subtypes: Luminal A, Luminal B, HER2-enriched, Basal- and Normal-like (Parker *et al.*, 2009). PAM50 was successfully used to assess both prognostic and predictive value to adjuvant hormonal therapy in breast cancer patients (Chia *et al.*, 2012). In Supplementary Table S2, we present the list of genes included in the commercial platforms that are currently used in therapeutic decisions in breast cancer: Prosigna/PAM50 (50 genes) and Oncotype (16 genes). Thus, throughout this article, we will refer to Prosigna and PAM50 indifferently. We also include in Supplementary Table S2 the list of genes developed in this work and used in our analysis (i.e. the signature of 34 genes). In our comparative study, when indicating Prosigna or PAM50, we used the expression of 49 encoding genes present in these platforms, excluding one gene (KNTC2, that encodes a kinetochore protein) because we had not signal for this gene in the expression data matrix of the two sets used in our study (microarrays or RNA-seq). In any case, we tested that the experimental results did not change by discarding one gene.

As a briefing or summary of the results of this study, we tried to answer three relevant questions: (i) Is there a small subset of genes that display their expression level associated with breast cancer prognosis in the same way as each of the three proteins regularly tested in the clinic by IHC?; (ii) Is there a robust and stable subset of gene survival markers that are able to improve the risk prediction and stratification provided by the gene sets considered in the commercial genomic platforms regularly used in the clinic to breast tumors?; (iii) Could some of these genes be considered novel prognostic markers because they better support the three IHC protein markers (ER, PR and HER2) that are regularly tested in the clinic to aid in therapeutic decisions?

### 3.1 Finding robust survival markers associated with the three standard IHC clinical markers used in BRCA

We identified a list of genes correlated with the three IHC standard tumor markers (ER, PR and HER2) most commonly used in the clinic to categorize breast cancer. To achieve this, we developed and applied Algorithm 1 based on an ensemble of elastic-net classifiers. In this algorithm, the number of bootstrap iterations $N_{B}$ were =100 and for the regularization term the α was =0.8. We selected a list of genes significantly associated with each of the three main clinical markers with stability index >0.10. In a further step, we carried out univariate and multivariate survival analysis of the genes using the methodology presented in Section 2.2. Finally, we selected for each clinical marker the genes more correlated with survival according to the log-rank statistics that were 16 genes for ER; 10 genes for PR (8 of them the same as ER); and 14 genes for HER2. The gene markers for HER2 did not show a highly significant association with survival (considering log-rank statistics), probably because most patients are treated with anti-HER2 drugs after diagnosis. Tables 3–5 show the genes selected, that in total are 32 distinct unique genes associated with ER, PR and HER2 with good stability. The genes, in alphabetical order by their gene symbols, are AGR3, CA12, CCDC170 (labeled C6orf97 in Tables 3 and 4), CDK12 (labeled CRKRS in Table 5), CNKSR1, CWC25, DNALI1, ERBB2, ESR1, GATA3, GFRA1, GRB7, KLC4, KMO, MED1, MIEN1 (labeled C17orf37 in Table 5), NANOS1, NAT1, NME3, PGAP3, PGR, PNMT, PSMD3, SIRT3, SLC15A2, SLC39A6, SOX11, STARD3, SUSD3, TBC1D9, TFF1 and ZNF552. Two extra genes that are standard indicators of cell proliferation were added to this list: AURKA and MKI67. In this way, we set up a final gene pool that includes 34 selected genes. Next, we checked whether the list of 34 selected genes was able to improve the prediction of IHC marker status made with individual genes (i.e. if the 34 genes could be better predicting whether the IHC markers are positive or negative in a given queried tumor). Table 2 shows the accuracy for the prediction of the ER, PR and HER2 status based on the signal of each single gene or based on the signal of the 34 genes. The first row corresponds to the 34-gene signature proposed in this study. The next rows show the accuracy based only on the gene that best represents the clinical marker: ESR1 gene for the ER, PGR gene for the PR and ERBB2 gene for the HER2. The 95% confidence intervals are provided to assess the significance of the differences among the prediction accuracy. These results show that the approach proposed in this article outperforms the predictors based on a single gene for all the three clinical markers considered. This suggests that the effect of these variables is better modeled by the cooperative work of several genes than by each single gene.

**Table 2.** Prediction accuracy for ER, PR, HER2 status, considering the 34 genes signature or only the expression of a single gene: ESR1, PGR or ERBB2 (maximum value = 1.0)

Predictor	ER	PR	HER2
34g signature	0.92 (0.89–0.95)	0.88 (0.85–0.91)	0.83 (0.79–0.87)
ESR1 gene	0.87 (0.83–0.90)	–	–
PGR gene	–	0.82 (0.78–0.86)	–
ERBB2 gene	–	–	0.75 (0.71–0.80)

*Note*: Confidence intervals (95%) are provided below in parentheses.

**Table 3.** Survival analysis (multivariate and univariate) of the list of selected genes associated with the status of the IHC marker ER: 16 genes included

gene Name	Univariate metrics			Multivariate	
	Log-rank *P*-value	$\beta_{U\mathrm{niCox}}$	$\beta_{U\mathrm{niCox}}$ *P*-value	$\beta_{M\mathrm{ultiCox}}$	$\sigma_{\beta_{M\mathrm{ultiCox}}}$
TBC1D9	0.00000057	−0.273	0.000067	−0.0461	0.0119
SUSD3	0.00000880	−0.298	0.000080	−0.0490	0.0146
SLC39A6	0.00006319	−0.225	0.001527	−0.0374	0.0127
GFRA1	0.00000189	−0.177	0.001640	−0.0260	0.0079
SOX11	0.00000027	0.154	0.003205	0.0250	0.0097
GATA3	0.00249397	−0.154	0.012805	−0.0254	0.0100
SLC15A2	0.00831951	0.316	0.017415	0.0779	0.0416
C6orf97	0.00029611	−0.244	0.017643	−0.0494	0.0213
NANOS1	0.00153739	0.153	0.019000	0.0264	0.0138
ZNF552	0.00003888	−0.182	0.019474	−0.0345	0.0156
ESR1	0.00057954	−0.239	0.030505	−0.0489	0.0218
NAT1	0.00459318	−0.098	0.032377	−0.0133	0.0061
NME3	0.00342702	−0.303	0.037352	−0.0799	0.0426
DNALI1	0.00320825	−0.112	0.068916	−0.0189	0.0109
AGR3	0.00628798	−0.056	0.070142	−0.0071	0.0042
CA12	0.00012777	−0.104	0.079446	−0.0167	0.0099

**Table 4.** Survival analysis (multivariate and univariate) of the list of selected genes associated with the status of the IHC marker PR: 10 genes included

Gene name	Univariate metrics			Multivariate	
	Log-rank *P*-value	$\beta_{U\mathrm{niCox}}$	$\beta_{U\mathrm{niCox}}$ *P*-value	$\beta_{M\mathrm{ultiCox}}$	$\sigma_{\beta_{M\mathrm{ultiCox}}}$
SUSD3	0.0000088	−0.298	0.00008	−0.0445	0.0133
GFRA1	0.0001724	−0.177	0.00164	−0.0233	0.0071
PGR	0.0001936	−0.161	0.01368	−0.0217	0.0100
C6orf97	0.0002847	−0.244	0.01764	−0.0456	0.0196
ESR1	0.0002989	−0.239	0.03050	−0.0452	0.0202
NAT1	0.0013054	−0.098	0.03238	−0.0119	0.0055
DNALI1	0.0023350	−0.112	0.06892	−0.0171	0.0099
AGR3	0.0011249	−0.056	0.07014	−0.0063	0.0037
CA12	0.0001278	−0.104	0.07945	−0.0151	0.0089
TFF1	0.0037668	−0.053	0.09463	−0.0064	0.0037

**Table 5.** Survival analysis (multivariate and univariate) of the list of selected genes associated with the status of the IHC marker HER2: 14 genes included

Gene name	Univariate metrics			Multivariate	
	Log-rank *P*-value	$\beta_{U\mathrm{niCox}}$	$\beta_{U\mathrm{niCox}}$ *P*-value	$\beta_{M\mathrm{ultiCox}}$	$\sigma_{\beta_{M\mathrm{ultiCox}}}$
PNMT	0.0155	0.140	0.049	0.0358	0.023
CWC25	0.1768	0.234	0.088	0.0706	0.059
C17orf37	0.0056	0.118	0.162	0.0258	0.023
SIRT3	0.0093	−0.371	0.207	−0.1940	0.133
MED1	0.3471	0.116	0.268	0.0301	0.036
ERBB2	0.0279	0.069	0.357	0.0149	0.018
KMO	0.2884	0.063	0.366	0.0152	0.017
PSMD3	0.0149	0.081	0.424	0.0167	0.033
GRB7	0.0244	0.049	0.511	0.0094	0.016
CRKRS	0.3292	0.069	0.556	0.0214	0.043
PGAP3	0.1891	0.046	0.599	0.0093	0.024
KLC4	0.1549	−0.053	0.867	−0.0399	0.203
STARD3	0.0476	0.017	0.869	0.0016	0.036
CNKSR1	0.8930	0.014	0.936	0.0037	0.074

Once we have a list of 34 gene markers associated with the IHC clinical status, we performed a full analysis of these genes with respect to tumor survival and risk prediction, using the univariate or multivariate methods presented in Section 2.2. Tables 3–5 provide the genes selected associated with the status of each one of the three IHC markers: ER, PR and HER2. The tables summarize the results using univariate and multivariate analysis. The Log-rank *P*-value is the *P*-value for the nonparametric log-rank test. The optimization procedure employed is similar to the Algorithm 2. This is an univariate test that evaluates the ability of a single gene to stratify a set of patients by the expression level in two groups of significantly different survival. The $\beta_{\text{UniCox}}$ is the estimation for the expected value of the regression coefficient in the univariate Cox regression model introduced in Section 2.2. The *P-value UniCox* is the Wald statistic *P*-value that evaluates if the regression coefficient is significantly different from zero. Significant *P*-values mean that there is an association between the gene expression and the risk. Therefore, the genes can be ranked according to their individual effect on the patient risk and survival considering the univariate metrics *P-value UniCox* or *Log-rank P-value*. Notice that as we have mentioned in Section 2.2 all the metrics have been computed using a bootstrap strategy to increase the robustness of the results.

Regarding the multivariate approach, the $\beta_{\mathrm{MultiCox}}$ is the estimation for the expected regression coefficient in the multivariate Cox regression model with $L_{1}$ norm penalization (introduced in Section 2.2). Larger absolute values correspond to genes that have strong impact on the patient risk. This metric takes into account the cooperative work between genes. The $\sigma_{\beta_{\mathrm{MultiCox}}}$ is the standard deviation of the regression coefficient. The standard deviation will help to determine if the $\left|\beta_{i}\right|$ are significantly greater than zero.

From the results presented in Table 3, we observed that the *P-value UniCox* for the ESR1 is significant and the $\beta_{\mathrm{UniCox}}$ <0 (negative), indicating that the over-expression of this gene reduces the risk in breast cancer. This is expected since the oncologists know that breast tumors that are ER positive have a better prognosis than tumors that are ER negative. Furthermore, the results in this table show that the expression of genes TBC1D9, SUSD3, CCDC170(C6orf97) and NME3 is also associated with low risk, and they show a stronger association with good survival than gene ESR1. Therefore, they can be proposed as new complementary markers to the ER clinical status. Finally, the genes found for HER2 did not show a highly significant association with survival, which may be due to the fact that these patients are usually treated with anti-HER2 drugs. However, the $\beta_{i}$ are often positive which is consistent with the empirical evidence that overexpression of HER2 increases patient risk.

### 3.2 Risk prediction of BRCA tumors based on gene expression signatures: 34 genes here proposed, Oncotype genes and Prosigna genes

A relevant application of the gene expression signatures measured in the breast tumors is to achieve an accurate prediction of the risk expected for each patient, and a consequent stratification of the tumors. To evaluate this, we have applied the multivariate Cox regression (as described in Section 2.2). We have compared the risk prediction based on the 34 genes signature proposed and the risk prediction and survival curves obtained using the same algorithm with the lists of genes that are included in two commercial genomic platforms that are already used in the clinic: Oncotype (including 16 genes) and Prosigna/PAM50 (including 49 genes). The lists indicating the names of all these genes are included in Supplementary Table S2. To check the generalization ability of these three gene signatures we tested them using an independent breast cancer dataset of 879 samples.

**Fig. 2.**
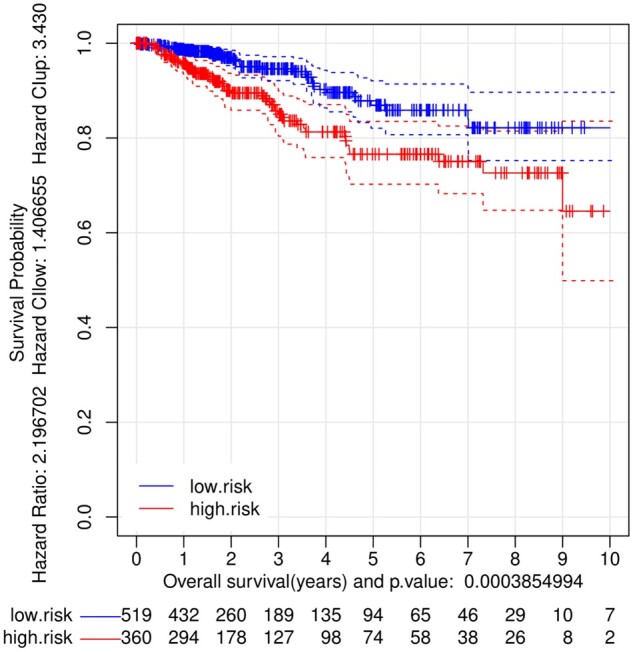
Kaplan–Meier curves (high-risk red versus low-risk blue) obtained with the 34 genes prognosis signature proposed in this work, tested here in the independent dataset of 879 breast cancer (BRCA) tumor samples (which includes patient survival information and gene expression data obtained with RNA-seq). The initial number of samples was splitted by the algorithm into: 519 low-risk samples and 360 high-risk samples; *P*-value = 0.00038

Figure 2 shows the Kaplan–Meier survival curves obtained after the splitting of the 879 tumors in two groups: one of low risk (that included 519 samples) and another of high risk (that included 360 samples). These two risk groups were obtained considering the expression values in the tumors of the 34 genes signature. The same analysis was done using the 16 genes signature of Oncotype and the 49 genes signature of Prosigna/PAM50. The corresponding Kaplan–Meier survival curves are included in Supplementary Figure S2a and b. We remark that the 34-gene signature proposed in this work generalizes very well over the validation dataset of 879 RNA-seq samples, that is completely independent to the first data set. Moreover, as indicated before, these three gene signatures were also compared with the 644 test samples of the first dataset and the corresponding Kaplan–Meier survival curves are presented in Supplementary Figure S1a–c.

Table 6 provides a summary of the results indicating the *log-rank statistic P-value* and the *hazard ratio* (HR) for the risk groups obtained based on each of the three signatures tested: the 34 genes signature and the ones including the genes used by Oncotype or by Prosigna/PAM50 platforms. The table shows that the signature of 34 genes performs significantly better than Prosigna/PAM50 and Oncotype, with the *log-rank statistic* and the HR significantly improved. This means that the risk prediction and the stratification of patients in two prognostic groups are significantly better considering the 34 genes signature. Since the results are obtained using an independent dataset, different from the one used to produce the signature, we can suggest that the gene expression signature found is quite stable and reproducible.

**Table 6.** BRCA patient stratification by risk based on the 34 genes signature here proposed or the signatures used by the genomic platforms Oncotype (16 genes) or Prosigna/PAM50 (49 genes)

Signature	*P*-value log-rank	HR	Confidence interval HR
34-gene signature	0.00038	2.20	1.41–3.43
Oncotype (16 g)	0.066	1.61	0.96–2.69
Prosigna/PAM50 (49 g)	0.035	1.60	1.03–2.49

*Note:* Results obtained with the breast cancer dataset of 879 samples.

Finally, several authors have studied the relationship among some survival markers discovered in this research and breast cancer. In particular, AGR3 (Garczyk *et al.*, 2015), CA12 (Li *et al.*, 2019), CCDC170 (C6orf97; Yamamoto-Ibusuki *et al.*, 2015), NDPKC (NME3; Wu *et al.*, 2020), PEPT2 (SLC15A2; El Ansari *et al.*, 2018), SUSD3 (Zhao *et al.*, 2016) and ZIP6 (SLC39A6; Althobiti *et al.*, 2021) have been linked to breast cancer in different studies and several of these genes have also been reported to interact or be associated with ER expression and function. Furthermore, as can be seen from the included references, several of these genes have been correlated with patient survival and prognosis in breast cancer studies. In some cases, upregulated expression of these genes was associated with prolonged survival and thus good prognosis, mainly in luminal breast cancer subtypes. This is the case of genes AGR3, CCDC170 and NDPKC. CA12 has also been correlated with a chemoresistance phenotype in cancer; in particular, silencing this gene has been shown to reverse paclitaxel sensitivity in drug-resistant breast cancer cells (Huang *et al.*, 2021). In this respect, the expression of multidrug resistance gene 1 (MDR1, also called ABCB1) in breast tumors has been associated with treatment and with a poor response to chemotherapy (Trock *et al.*, 1997).

With respect to genes CDK12 (CRKRS), CWC25 (CCDC49), GRB7, MIEN1 (C17orf37), PNMT and SIRT3, they have been shown to be linked to HER2 receptor and cancer (Oh *et al.*, 1999). Several of these genes have also been included in gene prognostic signatures published for HER2-positive breast cancer (Knauer *et al.*, 2010; Staaf *et al.*, 2010). In addition, the relationship of these genes with HER2 in breast cancer has already been demonstrated for several of them. For example, CDK12 positivity was correlated with HER2 positivity (Naidoo *et al.*, 2018); and MIEN1 overexpression facilitates migration, invasion and metastasis in breast cancer (Zhao *et al.*, 2017) and it is negatively correlated with disease free survival (Kushwaha *et al.*, 2019) mainly in HER2-positive subtypes.

## 4 Discussion

Accurate prediction of tumor risk based on easy-to-determine gene expression signatures associated with survival may be a critical step for the advance in breast cancer prognosis and treatment. Many gene survival signatures reported in the literature are unstable (Gong *et al.*, 2018; Haury *et al.*, 2011) and it is frequent that risk predictors provided by companies to physicians and oncologists lack transparent biological and biomolecular explanation or interpretation.

To improve the robustness of the survival gene signatures some authors have integrated gene expression data with gene interaction networks, protein–protein interaction networks or pathways information (Cun and Fröhlich, 2012; Das *et al.*, 2015). However, the integration of uncertain biological knowledge a priori can introduce a negative bias in the final model. Our approach is more related with ensemble methods (Gong *et al.*, 2018; Zhao *et al.*, 2011) that combine several predictors to improve the robustness of the selected signature by aggregating multiple gene lists. However, in some cases, the signatures proposed by multiple approaches do not allow biological interpretation in terms of the molecular markers that determine clinical outcomes (Manjang *et al.*, 2021). In this regard, genomic platforms such as Oncotype, Prosigna/PAM50 incorporate heuristic clinical knowledge to derive a more effective list of markers for therapeutic decisions. However, they work as black boxes because they do not reveal the clinical parameters considered, and therefore the correlation between the included individual gene markers and risk is unclear. Despite this, they have been successfully applied to predict risk and survival, providing a stratification of patients that helps the medical decision-making.

Our framework in this study identifies a set of survival markers that have a clear biological interpretation in terms of the three main biomolecular factors that determine the clinical prognosis in breast cancer: ER, PR and HER2. To ensure the stability and reproducibility of our results, we analyzed in this work two independent datasets with large number of samples. Furthermore, a robust feature selection strategy is developed based on bootstrap and ensemble methods. In this way, a 34 genes signature is proposed, improving significantly the risk prediction and patient stratification of the list of genes present in the commercial genomic platforms: Oncotype (16 genes) and Prosigna/PAM50 (49 genes).

## 5 Conclusion

The discovery and validation of survival biomarkers associated with a given phenotype or to a specific clinical variable is a critical step to achieve better disease prognosis. However, gene signatures proposed in the literature suffer frequently from the instability and irreproducibility problem and cannot be correlated well with clinical phenotypes.

In this article, we have developed a bioinformatic framework for the identification and validation of a robust and reproducible subset of gene survival markers that are related to clinical phenotypes in breast cancer. Algorithms are also introduced for the accurate risk prediction and optimal stratification of patients into risk groups. Combining several resources, we have integrated and analyzed two large breast cancer datasets that include genome-wide expression and survival data, as well as clinical pathological and phenotipic information. Furthermore, we have applied the framework in this dataset to discover a robust list of survival markers that correlate well with clinical IHC markers used in breast cancer. The results suggest that our signature of 34 genes improves the risk prediction and the patient stratification obtained using the genes included in two widely used genomic platforms.

Several of the gene markers found have been studied in the literature and are related to the prognosis of breast cancer or to some of the characteristics used in the categorization of breast tumors. Therefore, we report that some of the discovered survival-associated genes exhibit properties worthy of attention as novel alternative risk markers to standard IHC variables used in the clinic.

## Supplementary Material

vbad037_Supplementary_Data
