# Supplementary material for: Identification of a gene expression signature associated with breast cancer survival and risk that improves clinical genomic platforms
Source: Bioinform Adv. 2023 Mar 22;3(1):vbad037. doi: 10.1093/bioadv/vbad037 (PMC10122606; doi:10.1093/bioadv/vbad037)
Supplement: vbad037_Supplementary_Data [file vbad037_supplementary_data.pdf]

0.1 Supplementary Figures: Fig.1 and Fig.2

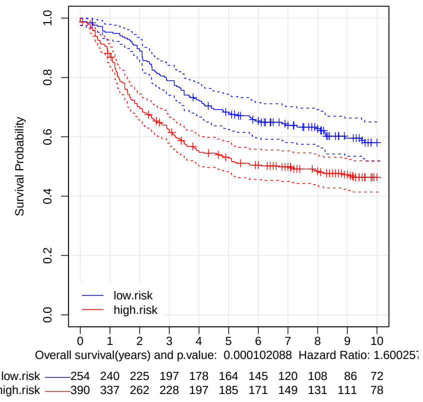

(a) Oncotype (p=0.00010)

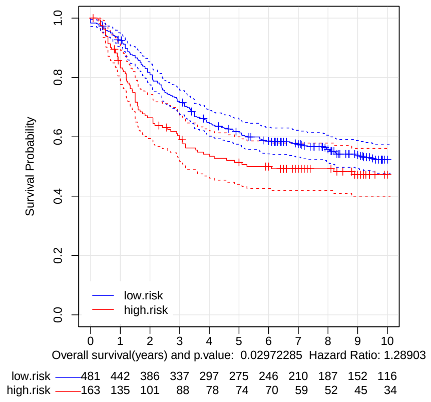

(b) Prosigna/PAM50 (p=0.02972)

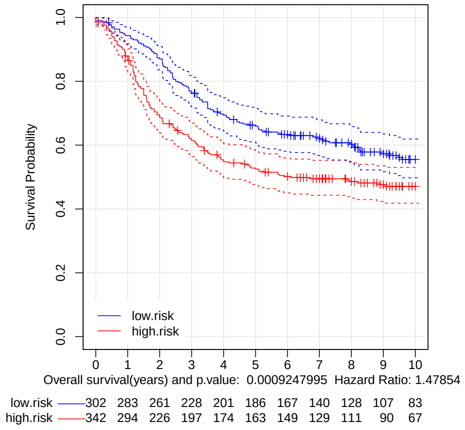

(c) 34 gene signature (p=0.00092)

Figure 1: Kaplan-Meier curves obtained for the subset of 644 samples (test set) from the first dataset of 1024 breast cancer tumors. The whole dataset includes survival information and genome-wide expression profiles obtained with high density expression microarrays. For a subset of 380 patients the 3 main clinical markers (ER, PR and HER2) have been determined by inmunohistochemistry. This subset has been used as training set to identify the genes associated to the main clinical markers and to train a multivariate risk predictor. The independent subset of 644 samples allow us to check the accuracy the 3 risk predictors: (a) based on the 16 genes of Oncotype; (b) based on the 49 genes of Prosigna/PAM50; and (c) based on the 34 genes of the signature that we developed in this work. Risk groups have been obtained by a risk threshold estimated by algorithm 2 over the training set.

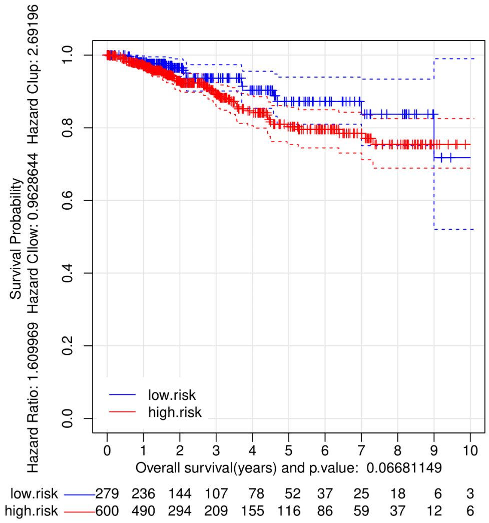

(a) Oncotype (p=0.0668)

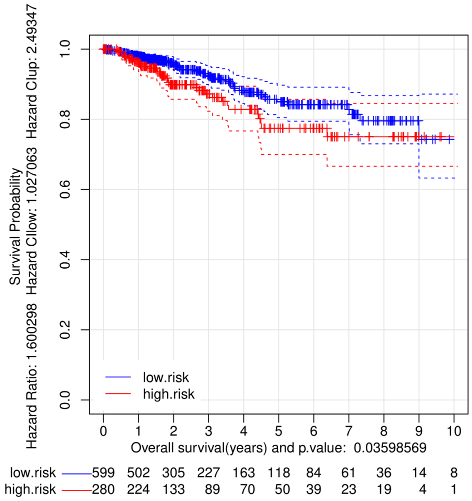

(b) Prosigna/PAM50 (p=0.0359)

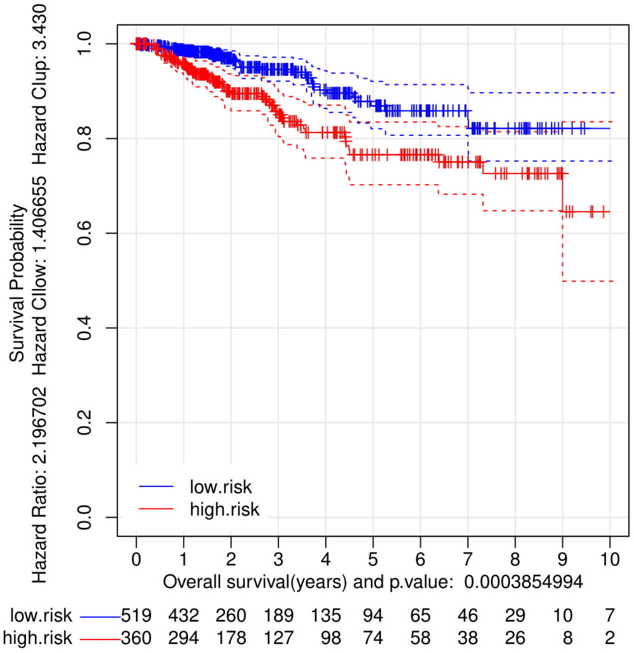

(c) 34 gene signature (p=0.00038)

Figure 2: Kaplan-Meier curves obtained for an independent data set that included 879 samples of breast cancer tumors with survival information and genome-wide expression data (obtained with RNA-seq). The lines in blue correspond to the samples with better survival (therefore to low.risk individuals) and the red lines to the samples with poor survival (therefore to high.risk individuals). Confidence intervals (95%) are marked with dotted lines. The Hazard Ratio (HR) associated with the risk is also calculated and presented within each plot. (a) Analysis done with Oncotype genes; (b) analysis done with Prosigna genes; (a) analysis done with our gene signature.

0.2 Suplementary Tables: Tab.1 and Tab.2

Table 1: GEO expression series integrated and normalized (using fRMA) in our large breast cancer data set; number of samples in each series; and number of batches per series.

Series	Samples	Number of batches
AffyBatchEsetGSE6532	87	1
AffyBatchEsetGSE6532	204	2
AffyBatchEsetGSE19615	115	1
AffyBatchEsetGSE19615	39	1
AffyBatchEsetGSE20685	327	3
AffyBatchEsetGSE21653	252	3

Table 2: List of genes included in the breast cancer genomic commercial platforms: Prosigna/PAM50 (50 genes) and Oncotype (16 genes); and list of genes included in the signature proposed in this paper (34 genes). In the experiments we have considered 49 genes for Prosigna due to the inability to find one gene in the expression platform used (gene KNTC2). However, we found that the experimental results do not change by removing just one gene. Notice that the overlapping between the different lists is small.

**Prosigna=PAM50(50g)**		**Oncotype(16g)**	**34 gene signature**	
(1) ACTR3B	(28) KRT17	(1) BAG1	(1) AGR3	(26) SIRT3
(2) ANLN	(29) KRT5	(2) BCL2	(2) AURKA	(27) SLC15A2
(3) BAG1	(30) MAPT	(3) BIRC5	(3) MIEN1	(28) SLC39A6
(4) BCL2	(31) MDM2	(4) CCNB1	(4) CCDC170	(29) SOX11
(5) BIRC5	(32) MELK	(5) CD68	(5) CA12	(30) STARD3
(6) BLVRA	(33) MIA	(6) CTSV	(6) CNKSR1	(31) SUSD3
(7) CCNB1	(34) MKI67	(7) ERBB2	(7) CDK12	(32) TBC1D9
(8) CCNE1	(35) MLPH	(8) ESR1	(8) CWC25	(33) TFF1
(9) CDC20	(36) MMP11	(9) GRB7	(9) DNALI1	(34) ZNF552
(10) CDC6	(37) MYBL2	(10) GSTM1	(10) ERBB2	
(11) CDCA1	(38) MYC	(11) MKI67	(11) ESR1	
(12) CDH3	(39) NAT1	(12) MMP11	(12) GATA3	
(13) CENPF	(40) ORC6L	(13) MYBL2	(13) GFRA1	
(14) CEP55	(41) PGR	(14) PGR	(14) GRB7	
(15) CXXC5	(42) PHGDH	(15) SCUBE2	(15) KLC4	
(16) EGFR	(43) PTTG1	(16) AURKA	(16) KMO	
(17) ERBB2	(44) RRM2		(17) MED1	
(18) ESR1	(45) SFRP1		(18) MKI67	
(19) EXO1	(46) SLC39A6		(19) NANOS1	
(20) FGFR4	(47) TMEM45B		(20) NAT1	
(21) FOXA1	(48) TYMS		(21) NME3	
(22) FOXC1	(49) UBE2C		(22) PGAP3	
(23) GPR160	(50) UBE2T		(23) PGR	
(24) GRB7			(24) PNMT	
(25) KIF2C			(25) PSMD3	
(26) KNTC2				
(27) KRT14				
